# RETRACTED: Vorotilo et al. Manufacturing of Conductive, Wear-Resistant Nanoreinforced Cu-Ti Alloys Using Partially Oxidized Electrolytic Copper Powder. *Nanomaterials* 2020, *10*, 1261

**DOI:** 10.3390/nano14161325

**Published:** 2024-08-07

**Authors:** Stepan Vorotilo, Pavel Alexandrovich Loginov, Alexandr Yuryevich Churyumov, Alexey Sergeevich Prosviryakov, Marina Yakovlevna Bychkova, Sergey Ivanovich Rupasov, Anton Sergeevich Orekhov, Philipp Vladimirovich Kiryukhantsev-Korneev, Evgeny Alexandrovich Levashov

**Affiliations:** 1 Scientific-Educational Center of SHS, National University of Science and Technology “MISiS”, Leninsky Prospect 4, 119049 Moscow, Russia; pavel.loginov.misis@list.ru (P.A.L.); bychkova@shs.misis.ru (M.Y.B.); vosapur@mail.ru (S.I.R.); kiruhancev-korneev@yandex.ru (P.V.K.-K.); levashov@shs.misis.ru (E.A.L.); 2 Department of Physical Metallurgy of Non-ferrous Metals, National University of Science and Technology “MISiS”, Leninsky Prospect 4, 119049 Moscow, Russia; churyumov@misis.ru (A.Y.C.); pro.alex@mail.ru (A.S.P.); 3 Laboratory of Electron Diffraction, Federal Scientific Research Centre “Crystallography and Photonics”, Russian Academy of Sciences, Leninsky Prospect 59, 119333 Moscow, Russia; orekhov.anton@gmail.com

The *Nanomaterials* Editorial Office retracts the article, “Manufacturing of Conductive, Wear-Resistant Nanoreinforced Cu-Ti Alloys Using Partially Oxidized Electrolytic Copper Powder” [1], cited above.

Following publication, concerns were brought to the attention of the Editorial Office regarding an overlap of images between this article and two other previously published articles [2,3] from different authorship groups.

Adhering to our complaint’s procedure, an investigation was conducted by the Editorial Office and Editorial Board that confirmed an overlap between the following:

- Figure 1, panel a in [1] and Figure 1 in [2],
- and Figure 1, panels b, c, and d in [1] and Figure 1, panels d, e, and f in [3].

While the authors cooperated with the investigation, they were unable to satisfactorily explain this overlap, nor were they able to provide raw images or data to validate the reliability of the findings presented. As a result, the Editorial Office and Editorial Board have decided to retract the paper as per MDPI’s retraction policy (https://www.mdpi.com/ethics#_bookmark30) and in line with the Committee on Publication Ethics retraction guidelines (https://publicationethics.org/retraction-guidelines). 

This retraction was approved by the Editor-in-Chief of the journal *Nanomaterials*.

The following authors disagree with the retraction of the paper: Pavel Alexandrovich Loginov, Marina Yakovlevna Bychkova, Sergey Ivanovich Rupasov, Philipp Vladimirovich Kiryukhantsev-Korneev, and Evgeny Alexandrovich Levashov. The remaining authors did not provide a comment on this decision.
